# Relationship between structural and functional changes in glaucomatous eyes: a multifocal electroretinogram study

**DOI:** 10.1186/s12886-021-02061-8

**Published:** 2021-08-21

**Authors:** Hiroki Tanaka, Kyoko Ishida, Kenji Ozawa, Takuma Ishihara, Akira Sawada, Kiyofumi Mochizuki, Tetsuya Yamamoto

**Affiliations:** 1 Department of Ophthalmology, Gifu University Graduate School of Medicine, 1-1 Yanagido, Gifu-shi, Gifu, 501-1194 Japan; 2 Department of Ophthalmology, Toho University Ohashi Medical Center, 2-22-36, Ohashi, Meguro-ku, Tokyo, 153-8515 Japan; 3 Gifu University Hospital, Innovative and Clinical Research Promotion Center, 1-1 Yanagido, Gifu-shi, Gifu, 501-1194 Japan

**Keywords:** Multifocal electroretinography, Optical coherence tomography, Humphrey field analyzer, Open angle glaucoma

## Abstract

**Background:**

The nasal to temporal amplitudes ratio (N/T) of multifocal electroretinography (mfERG) scans measured within 5° of the macula can be used to detect glaucomatous change. The photopic negative response (PhNR) of mfERG elicited by a circular stimulus centered on the fovea was significantly reduced in eyes with glaucoma. The PhNR to B-wave ratio (PhNR/B) is the optimal measure of the PhNR. However, clinical superiority for evaluating glaucoma patients has not been determined between N/T and PhNR/B yet.

**Methods:**

For morphological assessments, ganglion cell complex (GCC) in six regions and the average were measured by optical coherence tomography (OCT). For functional assessment, Humphrey visual fields (VF) with mean sensitivities (MT) and mfERG scans with parameters of N/T and the multifocal photopic negative response to B-wave ratio (mfPhNR/B) were measured. Sixty-nine eyes of 44 glaucoma patients were included and correlations between mfERG parameters and OCT or VF parameters were evaluated.

**Results:**

The mean age of patients was 59.4 years. The mean deviation for all eyes obtained with the VF 30–2 and VF 10–2 was − 7.00 and − 6.31 dB, respectively. Significant correlations between GCC thickness or VF parameter and the N/T were found, especially in the inferior and inforotemporal retinal areas corresponding to superior and superonasal VF sectors (GCC vs N/T; coefficient = − 7.916 and − 7.857, and MT vs N/T; coefficient = − 4.302 and − 4.437, in the inferior and inforotemporal retinal areas, respectively, all *p* values < 0.05). However, similar associations were not obtained between mfPhNR/B and OCT or VF parameters. The mfPhNR/B only in the inferotemporal sector was significantly correlated with the average thickness of GCC (coefficient = 4.823, *P* = 0.012).

**Conclusions:**

The N/T was correlated with GCC and VF in more numbers of measurement areas than the mfPhNR/B in the current study, however, a future study modifying the stimuli and amplitudes to obtain the spatial correspondence to OCT and VF measurement will be required to evaluate the value of mfERG.

**Supplementary Information:**

The online version contains supplementary material available at 10.1186/s12886-021-02061-8.

## Background

Glaucomatous optic neuropathy is associated with the loss of retinal ganglion cells (RGCs) and their axons. The photopic negative response (PhNR) of the full-field electroretinography (ERG) is a slow negative potential following the a- and b-waves that has been reported to originate primarily from the neural activities of the RGCs [1–3]. The amplitudes of the PhNR in focal ERG scans were significantly reduced in eyes with glaucoma [3–6]. However, the PhNR can be measured in several different ways, either as the negative trough following the b-wave or at a fixed time point [7–9]. Wu et al. recently reported that the PhNR to B-wave ratio (PhNR/B; B-wave amplitude defined as the a-wave trough to b-wave peak) exhibited the lowest magnitude of test–retest variability and concluded that PhNR/B was the optimal measure of the PhNR [10].

In earlier reports, the nasal to temporal amplitudes ratio (N/T) of the first slice of the second-order kernels of multifocal electroretinography (mfERG) scans measured within 5° of the macula was larger in glaucoma patients than in normal subjects. Further, a significant correlation was present between N/T and visual field (VF) parameters or the retinal thickness in the inferior quadrant in eyes with moderate glaucoma [11, 12].

These findings suggest that PhNR/B and N/T might be helpful in determining functional defects in patients with glaucoma and in diagnosing glaucoma. However, clinical superiority for evaluating glaucoma patients has not been determined between PhNR/B and N/T yet.

There are several reports available outlining direct comparisons among visual sensitivities determined by standard automated perimetry (SAP), structural parameters of the inner retina obtained by optical coherence tomography (OCT), and the amplitude of the PhNR/B or the N/T [4, 5, 13–15], but no reports especially conducting comparisons between N/T and the PhNR/B of mfERG scans (mfPhNR/B) in the same glaucoma patient have been published.

Thus, the aims of this study are to investigate the association between the morphological statuses of the macular region by OCT, the functional status including two mfERG parameters with N/T and mfRhNR/B, and the sensitivities of SAP and to determine the clinical superiority between mfPhNR/B and N/T in the same glaucoma patients.

## Methods

### Subjects

This was an observational cross-sectional study of patients treated at the Glaucoma Service of the Gifu University Hospital over a six-year period. We obtained written informed consent from all participants and all of the procedures conformed to the tenets of the Declaration of Helsinki. The Institutional Board of Research Associates of Gifu University Graduate School of Medicine approved our research protocols.

Open angle glaucoma (OAG) diagnoses were based on the presence of normal open-angle and glaucomatous optic nerve changes corresponding to VF defects. We classified the patients as having normal tension glaucoma (NTG) if none of the recorded intraocular pressures (IOPs) exceeded 21 mmHg in either eye at all examinations, while the remaining patients were classified as having primary OAG (POAG). Patients eligible for study inclusion had clinical diagnoses of POAG or NTG, a refractive spherical equivalent ranging between − 6.0 diopters (D) and + 3.0 D, and a best-corrected visual acuity (VA) of 0 logarithm of the minimum angle of resolution (logMAR) units or less.

We excluded patients with intraocular abnormalities other than glaucoma; those with significant cataracts that could induce refractive or VF errors; those with a history of any medication use that could affect the pupillary diameter, those with intraocular surgeries including laser therapy; and those with medical treatment changes in the interval among the VF tests, OCT examinations, and mfERG recordings. All examinations including VF, OCT, and mfERG were performed each other within 6 months. When both eyes met the criteria, two eyes of the patient were included in the study.

### OCT

Pupils were dilated with topical 0.5% tropicamide and 0.5% phenylephrine (Mydrin-P®; Santen Pharmaceutical, Osaka, Japan) before the OCT examinations with a Cirrus high-definition OCT (HD-OCT) 4000 instrument (Carl Zeiss Meditec, Jena, Germany). The software automatically collected measurements of the peripapillary retinal nerve fiber layer (RNFL) with a diameter of 3.46 mm consisting of 256 A-scans centered on the optic disc. We obtained the average thickness of the circumpapillary RNFL (cpRNFL), then used the Macula Cube 200 × 200 and Ganglion Cell Analysis (GCA) programs to collect additional data in glaucoma patients as follows.

**Fig. 1:**
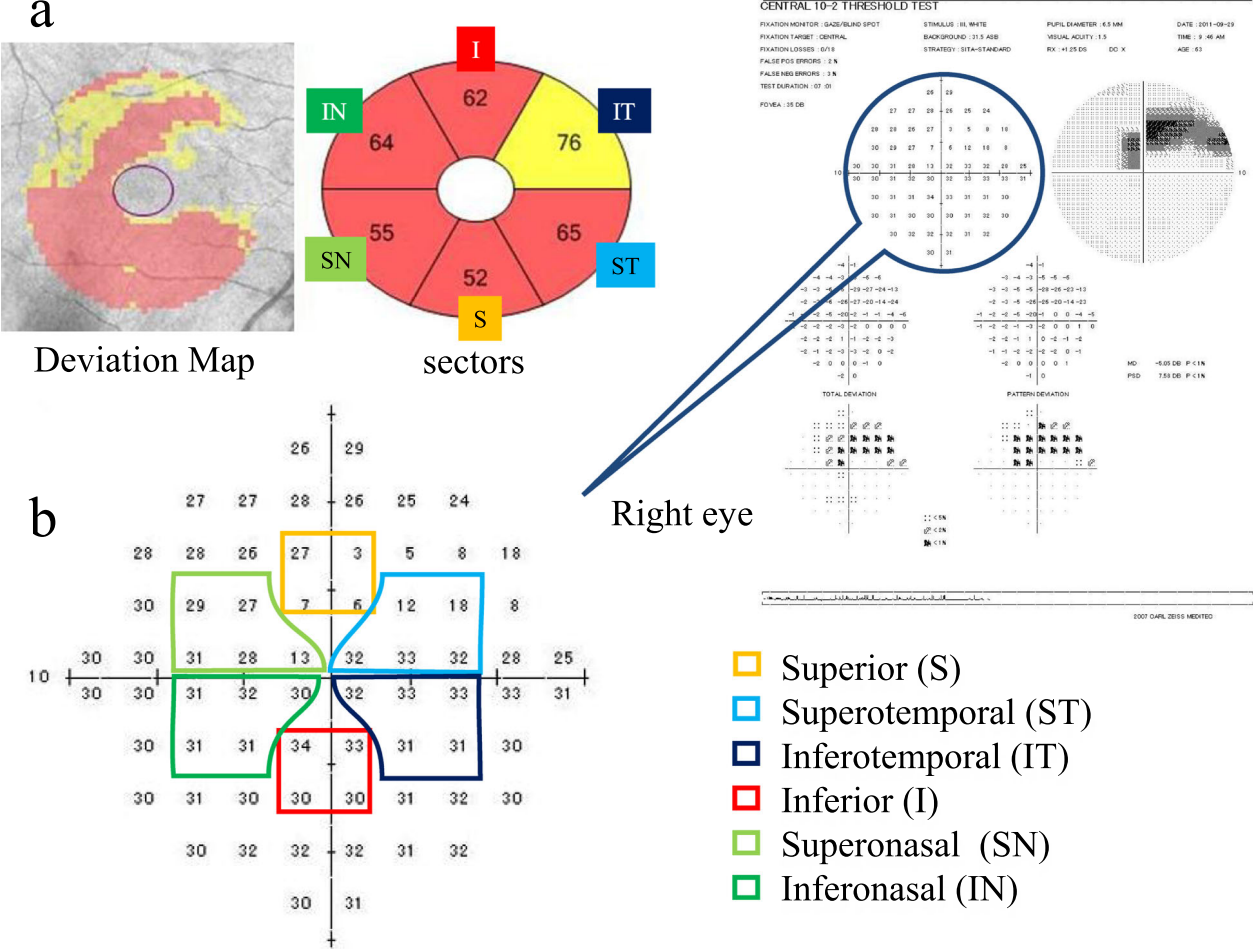
Association between optical coherence tomography (OCT) thickness and Humphrey Field Analyzer Central 10–2 program (HFA10–2). **a** The macular thickness is measured by OCT between two concentric circles of 2- and 6-mm diameters. **b** The findings of the HFA10–2 in right eye of glaucoma patient. Based on the retinal ganglion cell displacement [16], we classified the stimulus points on the HFA10–2 corresponding to the six sectors of the ganglion cell and inner plexiform layer (GCIPL) measurement ellipse into 6 groups (i.e., superior, superotemporal, inferotemporal, inferior, superonasal, and inferonasal sectors)

The macular cube scan generated one set of 200 horizontal B-scans, each composed of 200 A-scans centered on a 6- × 6-mm macular region. The built-in GCA algorithm (Cirrus H-OCT software, version 6.0) measured the thicknesses of the macular RNFL (mRNFL) and ganglion cell-inner plexiform layer (GCIPL) within a 6- × 6- × 2-mm cube in an elliptical annulus around the fovea. By using the GCA algorithm, the GCIPL thickness was calculated automatically as the distance from the outer boundary of the RNFL to the outer boundary of the inner plexiform layer (IPL) and stratified such as global and sectoral values (i.e., superonasal, superior, superotemporal, inferotemporal, inferior, and inferonasal sectors). All of the sectorial thickness obtained with OCT were shown based on the corresponding VF sectors. (Fig. 1a and b). We also measured the mRNFL thickness as the distance between the internal limiting membrane and the outer boundary of the RNFL and calculated the same six sectorial values. Ganglion cell complex (GCC) was measured as the value which added mRNFL with GCIPL and we also calculated the six sectorial values of GCC, similarly.

We only incorporated OCT images with a high quality of signal strength greater than 7/10 in the analysis.

### VF testing

All glaucoma participants underwent perimetric examinations using the Humphrey Field Analyzer (HFA) (750 I series; Carl Zeiss Meditec, Jena, Germany) with the Central 30–2 (HFA 30–2) and the Central 10–2 programs (HFA 10–2) using the Swedish Interactive Threshold Algorithm. We identified glaucomatous VF defects by the presence of three or more significant (*P* < 0.05) non–edge-contiguous points, with at least one point located at the *P* < 0.01 level in the pattern deviation plot along with grading outside the normal limits in the glaucoma hemifield test. VF tests were considered reliable when false-negative responses were less than 15%, false-positive responses were less than 15% and fixation losses were less than 20%. Based on the report of RGC displacement, we classified the stimulus points on the HFA 10–2 corresponding to the six sectors of the GCIPL measurement ellipse into six groups (Fig. 1b) [16]. We averaged the thresholds of each sector on the SAP.

### mfERG scans

All glaucoma patients underwent mfERG. We used the Visual Evoked Response Imaging System Science (VERIS) 5.1.10× (Electro-Diagnostic Imaging, Milpitas, CA, USA) to record mERG scans according to a published method [11, 12, 14]. After pupils were dilated to at least 8 mm in diameter with Mydrin-P®, we placed a bipolar contact lens electrode (Mayo, Inazawa, Japan) on the anesthetized (oxybuprocaine hydrochloride, Benoxil®; Santen Pharmaceutical, Osaka, Japan) cornea. We covered the contralateral eye, and then applied hydroxyethylcellulose gel (Scopisol®; Senju Pharmaceutical, Osaka, Japan) to the cornea to protect it from dehydration and to achieve good electrical contact between the electrodes and the cornea. We attached a gold-cup electrode to the right earlobe as a ground electrode. We then carried out refractions to elucidate the patients’ best VA for the stimulus viewing distance. Next, we adjusted the viewing distance to compensate for changes in the retinal image size due to the refractive lens used. During the mfERG recordings, the subjects sat with their chin and forehead tightly fixed. We instructed the subjects to fixate on a point at the center of the cathode ray tube (CRT) monitor while the eyes were being stimulated. The distance from the tested eye to the CRT monitor was 33 cm at zero diopters. The amplitudes of the mfERG were expressed as the response density, nV/deg^2^, or μV, representing the amplitudes as a function of the stimulus area.

#### P1 component of the first slice of second-order kernels of mfERG scans

**Fig. 2:**
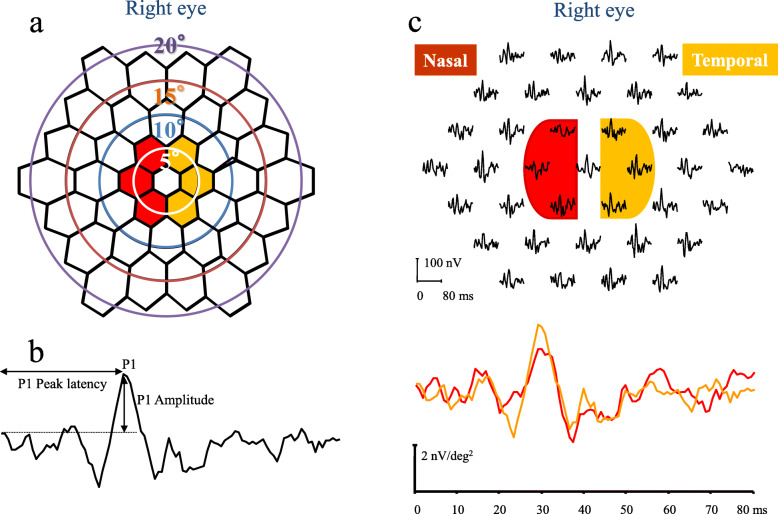
The stimulus array and the nasal to temporal amplitudes ratio obtained from multifocal electroretinograms (mfERGs). **a** The pattern of the 37-hexagon stimulus array with circles indicating radii of 5°, 10°, 15° and 20°in right eye. **b** The measurements of the first slice of the second-order kernels obtained from multifocal electroretinograms (mfERGs). The first positive peak, P1 amplitude was measured. **c** We separated the hexagons and averaged them according to the temporal (orange color) nasal (red color) hemispheres. We used the ratio of the amplitudes of the mfERGs of the nasal to the temporal hemisphere within the central 5° (N/T) to evaluate the asymmetry

The visual stimuli consisted of 37 hexagons that were displayed on a monochrome computer monitor (QB1781; Chuomusen, Tokyo, Japan) (Fig. 2a). The stimulus array subtended a visual angle of 50° by 40°. Each hexagonal element of the stimulus was independently alternated between black (5 cd/m^2^) and white (200 cd/m^2^; contrast: 95.1%) at a frame rate of 75 Hz according to a binary m-sequence. We set the bandpass filters at 10 to 300 Hz. We monitored the positions of the eyes during the recordings through the VERIS recording window. Each recording lasted approximately 4 mins, and we discarded segments with eye movements or blinking artifacts and recorded them again. We applied an artifact elimination technique once, with no spatial smoothing [17]. We studied the amplitudes of the first positive peak, P1 (Fig. 2b). The P1 amplitudes of the first slice of the second-order kernel responses were measured according to a published method [11, 12, 14].

#### Nasal to temporal amplitude ratio analyses of mfERG scans

The mfERG scans elicited by the 37-hexagon stimulus array within a circle of a 5° radius are shown in Fig. 2a. We compared the summed mfERG scans from the central 5° of the nasal VF (i.e., temporal hemisphere of the central 5° retinal area; red color in Fig. 2a and c) with those in the temporal hemisphere of the central 5° VF (i.e., nasal hemisphere of the central 5° retinal area; orange color in Fig. 2a and c). We calculated the N/T—namely, the ratio of the mfERG P1 amplitudes of the first slice of the second-order kernel (Fig. 2b)—in the nasal hemisphere of the VF (i.e., temporal hemisphere of the retina) and compared with that in the temporal hemisphere of the VF (i.e., nasal hemisphere of the retina) in the central 5°. We also calculated the correlations between the thresholds obtained from the corresponding VF area, OCT parameters, and the N/T [18, 19].

#### Multifocal photopic negative response

**Fig. 3:**
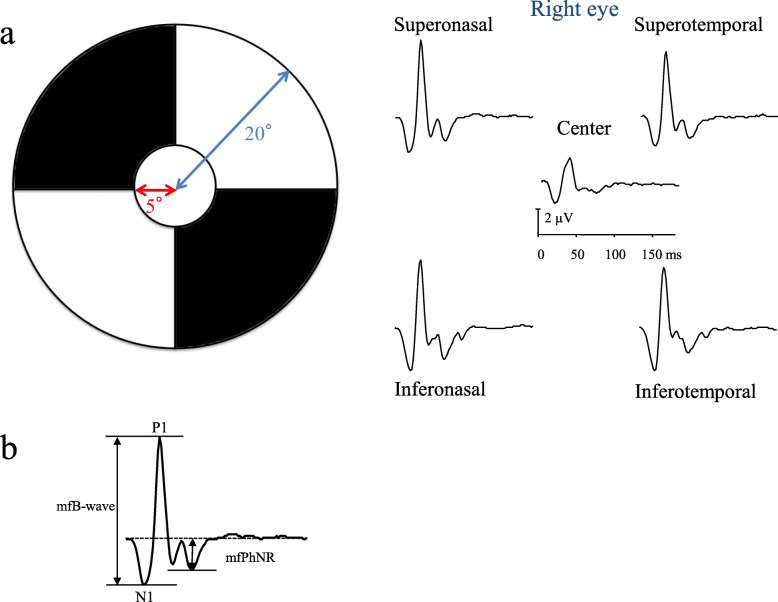
The stimulus patterns and the multifocal photopic negative response (mfPhNR). **a** The stimulus patterns (a circular stimulus with a 5° radius centered on the fovea and a quarter of an annulus placed in the superotemporal, superonasal, inferotemporal, and inferonasal regions around the fovea) were used to elicit mfERGs. The radius of the inner border of the annulus was 5° and that of the outer border was 20°. **b** The representative waveforms of the mfERGs recoded from five sectors in right eye of a patient with glaucoma. We measured the multifocal B-wave (mfB-wave; P1–N1) from the first negative trough to the peak of the following positive wave. The mfPhNR was measured from the baseline to the negative trough at more than 70 ms from the stimulus onset

The mfPhNRs were elicited by a circular stimulus with a 5° radius centered on the fovea and by a quarter of an annulus placed in the superotemporal, superonasal, inferotemporal, and inferonasal regions around the fovea (Fig. 3a). The radius of the inner border of the annulus was 5° and that of the outer border was 20°. White (200 cd/m^2^) or black (5 cd/m^2^) elements were presented in a pseudorandom binary m-sequence at a frequency of 37.5 Hz. Each recording lasted approximately 2 mins. A steady background surrounded the stimulus field. We measured the multifocal a-wave amplitude from the baseline to the trough of the first negative response and the multifocal B-wave (mfB-wave; P1–N1) from the first negative trough to the peak of the following positive wave [20]. The PhNR was measured from the baseline to the negative trough at more than 70 ms from the stimulus onset (Fig. 3b) [6].

#### mfPhNR/B analyses

**Fig. 4:**
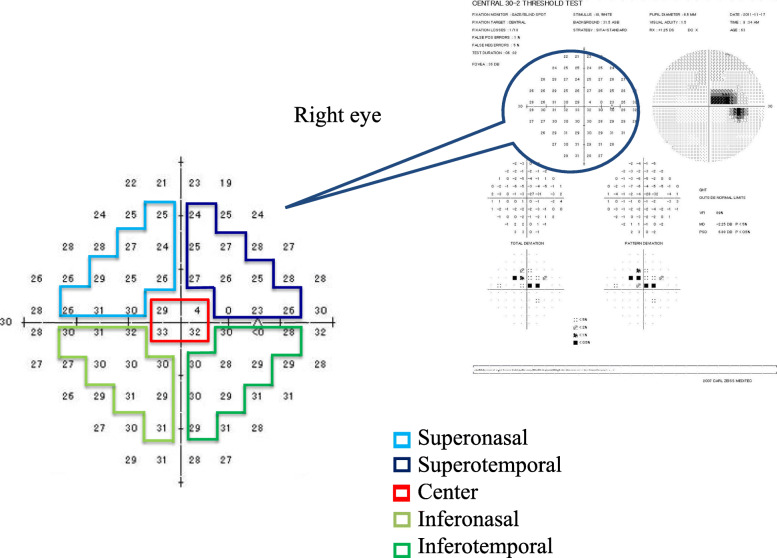
Humphrey Field Analyzer Central 30–2 program (HFA 30–2) corresponding to the mfPhNR regions. (see Fig. 3). To compare the multifocal photopic negative response to B-wave ratio (mfPhNR/B) with the corresponding visual field findings, we measured the thresholds with the HFA 30–2 and averaged for the same sectors (i.e., superonasal, superotemporal, center, inferonasal, and inferotemporal) according to the distance from the macula within the central 20°

We calculated the amplitudes of the mfPhNR/B in each sector. To compare the mfPhNR/B with the corresponding VF findings, we measured the thresholds with the HFA 30–2 and averaged for the same sectors according to the distance from the macula within the central 20° (Fig. 4). We also calculated the correlations between the thresholds of the corresponding VF area, the mfPhNR/B, and OCT parameters [18, 19].

### Statistical analyses

The demographic data of glaucoma patients was summarized using mean ± SD with range for continuous variables and frequencies for categorical variables. To assess the relationship between parameters, we used the multivariable regression model with Huber-White robust sandwich estimator because the data encompassed repeated observations (right and left eyes) in the same patient. The multivariable regression model was adjusted for covariates including age and spherical equivalent as potential confounder. Moreover, we used the restricted cubic splines to allow for nonlinear associations between parameters. Because nonlinearity was taken into account in the parameters, the coefficients for changes from the 25th percentile to the 75th percentile were reported as representative. A two-sided significance level was 0.05. We accepted an association only if the *p*-value of the statistical test of the regression coefficient was below the significance level. All analyses were performed using R software (www.r-project.org).

## Results

### Demographic data of glaucoma patients

The demographics of the 69 eyes of 44 patients with OAG included in this study are presented in Table 1. The mean patient age was 59.4 years, while the mean IOP for all eyes was 13.8 mmHg. Seventeen eyes had POAG and 52 had NTG. Thirty-two eyes were using topical antiglaucoma medications. The mean deviation (MD) for all eyes obtained with the HFA 30–2 was − 7.00 dB and the pattern standard deviation (PSD) was 9.01 dB. Similarly, the MD with the HFA 10–2 was − 6.31 dB and the average PSD was 7.85 dB. Forty eyes were early, 13 were middle-stage, and 16 were advanced glaucoma. The mean cpRNFL, GCIPL, mRNFL, and GCC was 69.8, 68.9, 26.7, and 95.6 μm, respectively.

**Table 1:** The Demographic data of glaucoma patients

Variable		*N* = 44
Gender (male/female)		22 eyes/ 22 eyes
Eye (Right/Left)		32 eyes/ 37 eyes
Age [years]		59.4 ± 11.8 (35 ~ 78)
Type of Glaucoma (POAG/NTG)		17eyes/ 52eyes
Corrected Visual acuity (Log MAR)		−0.13 ± 0.06(− 0.18 ~ 0.00)
Spherical Equivalent [Diopters]		−1.94 ± 2.35(− 5.88 ~ + 3.00)
Intraocular Pressure [mmHg]		13.8 ± 2.8(7 ~ 21)
Medication (With/Without)		32/37
mfPhNR/B	Center	0.32 ± 0.10 (0.14 ~ 0.65)
	Superotemporal	0.21 ± 0.09 (0.07 ~ 0.69)
	Superonasal	0.21 ± 0.06 (0.10 ~ 0.41)
	Inferonasal	0.25 ± 0.07 (0.12 ~ 0.42)
	Inferotemporal	0.25 ± 0.07 (0.12 ~ 0.47)
N/T		0.83 ± 0.37 (0.11 ~ 2.50)
HFA Central 30–2 Program		
Mean Deviation [dB] (Early:> − 6/Moderate:-12≦ ≦ − 6/Advanced:<− 12)		−7.00 ± 7.34(− 29.92 ~ 2.06) 40/13/16
Pattern Standard Deviation [dB]		9.01 ± 5.23 (1.61 ~ 18.76)
Mean Threshold [dB] / Total Deviation	Center	27.1 ± 7.0 (9.8 ~ 35.5) / -6.0 ± 6.8 (− 25.3 ~ 2.3)
	Superotemporal	19.8 ± 8.8 (2.2 ~ 32.4) / -8.0 ± 9.2 (− 29.9 ~ 2.8)
	Superonasal	21.1 ± 11.6 (0.0 ~ 33.1) / -9.3 ± 12.1 (− 32.0 ~ 2.9)
	Inferonasal	22.6 ± 10.4 (0.0 ~ 33.4) / -8.7 ± 10.9 (− 33.1 ~ 2.8)
	Inferotemporal	23.8 ± 6.3 (0.0 ~ 30.6) / -4.3 ± 7.1 (− 32.6 ~ 2.0)
HFA Central 10–2 Program		
Mean Deviation [dB]		−6.31 ± 6.91(− 27.42 ~ 2.07)
Pattern Standard Deviation [dB]		7.85 ± 5.66 (0.98 ~ 17.19)
Mean Threshold [dB]/Total Deviation	Superotemporal	29.5 ± 7.4 (0 ~ 37.2) / -2.7 ± 6.1 (− 31.8 ~ 2.6)
	Superior	22.9 ± 13.3 (0 ~ 36.8) / -10.0 ± 13.6 (− 35.5 ~ 3.0)
	Superonasal	25.1 ± 12.1 (0.2 ~ 36.8) / -9.5 ± 12.6 (− 35.6 ~ 3.2)
	Inferonasal	30.9 ± 6.8 (5.42 ~ 37.2) / -3.3 ± 7.0 (− 29.6 ~ 3.2)
	Inferior	31.1 ± 6.0 (6.5 ~ 36.5) / -2.1 ± 5.7 (− 28.3 ~ 2.0)
	Inferotemporal	32.5 ± 4.0 (10.2 ~ 36.2) / -0.4 ± 2.1 (− 7.0 ~ 3.0)
Circumpapillary retinal nerve fiber layer thickness [μm]		69.8 ± 12.3(46 ~ 100)
OCT GCIPL thickness [μm]/ mRNFL thickness [μm]/ GCC thickness [μm]	Average	68.9 ± 8.1(47 ~ 84)/ 26.7 ± 5.2(12 ~ 40)/ 95.6 ± 12.7(62 ~ 121)
	Superotemporal	71.2 ± 10.4(38 ~ 90)/ 32.7 ± 8.6(10 ~ 47)/ 103.9 ± 17.4(60 ~ 132)
	Superior	64.6 ± 10.5(46 ~ 87)/ 25.6 ± 10.5(10 ~ 45)/ 90.2 ± 20.3(60 ~ 127)
	Superonasal	63.1 ± 11.5(46 ~ 87)/ 16.9 ± 6.1 (7 ~ 28)/ 80.0 ± 17.0(57 ~ 112)
	Inferonasal	68.4 ± 9.4(49 ~ 88)/ 18.7 ± 4.7 (7 ~ 29)/ 87.1 ± 13.4(58 ~ 112)
	Inferior	70.9 ± 10.3(33 ~ 88)/ 31.1 ± 6.4(14 ~ 46)/ 101.9 ± 15.8(55 ~ 134)
	Inferotemporal	75.2 ± 10.1(38 ~ 91)/ 35.0 ± 6.0(17 ~ 52)/ 110.1 ± 14.8(67 ~ 135)

*POAG* primary open angle glaucoma, *NTG* normal tension glaucoma, *LogMAR* Logarithm of the minimum angle of resolution, *mfPhNR/B* multifocal photopic negative response to multifocal B-wave ratio, *N/T* nasal to temporal amplitude ratio, *HFA* Humphrey Field Analyzer, *GCIPL* Ganglion cell-inner plexiform layer, *mRNFL* Macular retinal nerve fiber layer, *GCC* Ganglion cell complex, Values are mean ± standard deviation (range)

### Correlation between the N/T of mfERG, the macula thickness of OCT, and the mean thresholds and the total deviation of HFA 10–2 in each sector in glaucoma patients

The N/T was significantly correlated with the average thickness of GCIPL (coefficient = − 3.455, *P* = 0.009) (Table 2). The N/T was also significantly correlated with the thickness of GCIPL in superotemporal, superior, superonasal, and inferotemporal sectors, which corresponded to inferonasal, inferior, inferotemporal, and superonasal sectors of the retina (coefficient = − 4.767, *P* = 0.003, coefficient = − 3.461, *P* = 0.014, coefficient = − 5.311, *P* = 0.001, and coefficient = − 3.027, *P* = 0.032, respectively).

**Table 2:** Correlation between the N/T of mfERG, the macula thickness of OCT, and the mean thresholds and the total deviation of HFA 10–2 in each sector in glaucoma patients

OCT and HFA		vs N/T (IQR: 0.59–1.024)			
Parameter	Sectors	Coefficient	95% LCL	95% UCL	*P*-value
GCIPL of OCT	Average	−3.455	−5.896	−1.014	**0.009**
	Superotemporal	−4.767	−7.592	−1.942	**0.003**
	Superior	−3.461	−6.021	− 0.901	**0.014**
	Superonasal	−5.311	−8.19	−2.433	**0.001**
	Inferonasal	−2.349	−7.199	2.501	0.578
	Inferior	−2.134	−6.211	1.943	0.522
	Inferotemporal	−3.027	−6.023	− 0.032	**0.032**
	SN + IN/ST + IT	−0.001	− 0.031	0.029	0.639
mRNFL of OCT	Average	−2.354	−4.136	−0.572	**0.034**
	Superotemporal	−3.665	−6.407	−0.923	**0.026**
	Superior	−4.455	−7.321	−1.589	**0.008**
	Superonasal	−2.546	−4.118	−0.973	**0.007**
	Inferonasal	0.000	−2.590	2.591	0.775
	Inferior	−1.182	−4.261	1.897	0.624
	Inferotemporal	−2.706	−5.414	0.002	0.132
	SN + IN/ST + IT	0.015	−0.016	0.045	0.632
GCC of OCT	Average	−5.809	−9.928	−1.691	**0.017**
	Superotemporal	−8.432	−13.613	−3.252	**0.007**
	Superior	−7.916	−13.221	−2.61	**0.008**
	Superonasal	−7.857	−12.041	−3.673	**0.001**
	Inferonasal	−2.349	−9.577	4.88	0.810
	Inferior	−3.316	−10.332	3.7	0.642
	Inferotemporal	−5.733	−10.939	−0.527	0.055
	SN + IN/ST + IT	0.008	−0.016	0.033	0.735
Mean thresholds of HFA 10–2	Mean Deviation	−2.089	−4.100	−0.077	0.069
	Superotemporal	−1.759	−4.173	0.656	0.231
	Superior	−4.302	−7.772	−0.833	**0.045**
	Superonasal	−4.437	−7.652	−1.223	**0.020**
	Inferonasal	0.780	−1.728	3.287	0.425
	Inferior	−0.606	−2.360	1.149	0.773
	Inferotemporal	−0.864	−2.433	0.706	**0.026**
	SN + IN/ST + IT	0.033	−0.104	0.169	0.083
Total deviation of HFA 10–2	Superotemporal	−1.511	−3.588	0.567	0.097
	Superior	−4.329	−7.861	− 0.797	0.051
	Superonasal	−4.823	−8.172	−1.475	**0.018**
	Inferonasal	0.356	−2.525	3.236	0.604
	Inferior	−0.282	−1.932	1.369	0.943
	Inferotemporal	−0.219	−1.012	0.573	0.569
	SN + IN/ST + IT	−0.890	−6.775	4.994	0.802

Coefficient represents the increase in the value of parameter in each sector when N/T of mfERG changes by 75th percentile from 25th percentile. All multivariable regression models were adjusted for age and spherical equivalent

*N/T* nasal to temporal amplitude ratio, *mfERG* multifocal electroretinogram, *OCT* Optical coherence tomopraphy, *HFA 10–2* Humphrey Field Analyzer Program Central 10–2, *IQR* interquartile range (25th percentile – 75th percentile), *LCL* lower confidence limit, *UCL* upper confidence limit, *GCIPL* Ganglion cell-inner plexiform layer, *SN* Superonasal, *IN* Inferonasal, *ST* Superotemporal, *IT* Inferotemporal, *mRNFL* Macular retinal nerve fiber layer, *GCC* Ganglion cell complex

For mRNFL, the N/T was significantly correlated with the average thickness of mRNFL (coefficient = − 2.354, *P* = 0.034), and with the thickness of mRNFL in superotemporal, superior, and superonasal sectors, which corresponded to inferonasal, inferior, and inferotemporal sectors of the retina (coefficient = − 3.665, *P* = 0.026, coefficient = − 4.455, *P* = 0.008, coefficient = − 2.546, *P* = 0.007, respectively).

For GCC, the N/T was significantly correlated with the average thickness of GCC (coefficient = − 5.809, *P* = 0.017), and with the thickness of GCC in superotemporal, superior, and superonasal sectors, which corresponded to inferonasal, inferior, and inferotemporal sectors of the retina (coefficient = − 8.432, P = 0.007, coefficient = − 7.916, P = 0.008, coefficient = − 7.857, P = 0.001, respectively).

For VF, the N/T was significantly correlated with MT in superior, superonasal, and inferotemporal sectors (coefficient = − 4.302, *P* = 0.045, coefficient = − 4.437, *P* = 0.020, and coefficient = − 0.864, P = 0.026, respectively). The N/T was also significantly correlated with TD in superonasal sector (coefficient = − 4.823, *P* = 0.018).

### The mfPhNR/B of mfERG, the mean threshold and total deviation of HFA 30–2, and the correlations between them in each sector in glaucoma patients

The mfPhNR/B was significantly negatively correlated with the MT and the total deviation in the superotemporal sector (coefficient = − 1.632, *P* = 0.013, and coefficient = − 1.701, P = 0.018, respectively) (Table 3).

**Table 3:** The mfPhNR/B of mfERG, the mean threshold and the total deviation of HFA 30–2, and the correlations between them in each sector in glaucoma patients

HFA 30–2		vs mfPhNR/B				
Parameter	Sectors	IQR	Coefficient	95% LCL	95% UCL	*P*-value
Mean Threshold [dB]	Center	0.269–0.385	−0.559	−2.553	1.435	0.839
	Superotemporal	0.160–0.261	−1.632	−3.028	−0.235	**0.013**
	Superonasal	0.160–0.237	−0.271	−2.654	2.112	0.684
	Inferonasal	0.193–0.299	1.124	−1.846	4.094	0.640
	Inferotemporal	0.194–0.317	0.221	−1.270	1.713	0.643
Total Deviation	Center	0.269–0.385	−0.238	−1.984	1.508	0.836
	Superotemporal	0.160–0.261	−1.701	−3.145	−0.258	**0.018**
	Superonasal	0.160–0.237	−0.368	−2.755	2.019	0.720
	Inferonasal	0.193–0.299	1.118	−2.013	4.250	0.717
	Inferotemporal	0.194–0.317	0.211	−1.481	1.904	0.562

Coefficient in each sector represents the increase in the value of parameter of HFA 30–2 when mfPhNR/B changes by 75th percentile from 25th percentile. All multivariable regression models were adjusted for age and spherical equivalent

*mfPhNR/B* multifocal photopic negative response to multifocal B-wave ratio, *mfERG* multifocal electroretinogram, *HFA 30–2* Humphrey Visual Field Analyzer Program Central 30–2, *IQR* interquartile range (25th percentile – 75th percentile), *LCL* lower confidence limit, *UCL* upper confidence limit

### Correlations between the mfPhNR/B of mfERG in each sector and the average thickness of the GCIPL, mRNFL, and GCC of OCT in glaucoma patients

The mfPhNR/B in the superotemporal and inferotemporal sector was significantly correlated with the average thickness of GCIPL (coefficient = − 0.025, *P* = 0.033 and coefficient = 2.459, *P* = 0.042, respectively) (Table 4).

**Table 4:** Correlations between the mfPhNR/B of mfERG in each sector and the average thickness of the GCIPL, mRNFL, and GCC of OCT in glaucoma patients

Sectors	IQR	Coefficient	95% LCL	95% UCL	*P*-value
mfPhNR/B	vs GCIPL average				
Center	0.259–0.385	0.839	−1.456	3.133	0.739
Superotemporal	0.151–0.261	−0.025	−3.793	3.743	**0.033**
Superonasal	0.160–0.240	1.013	−1.956	3.982	0.649
Inferonasal	0.192–0.306	1.096	−1.831	4.024	0.726
Inferotemporal	0.194–0.304	2.459	−0.791	5.71	**0.042**
	vs mRNFL average				
Center	0.259–0.385	0.863	−0.720	2.446	0.551
Superotemporal	0.151–0.261	1.04	−1.443	3.523	0.228
Superonasal	0.160–0.240	1.283	−0.919	3.485	0.483
Inferonasal	0.192–0.306	1.492	−0.414	3.399	0.301
Inferotemporal	0.194–0.304	2.363	0.416	4.31	**0.003**
	vs GCC average				
Center	0.259–0.385	1.701	−2.049	5.451	0.650
Superotemporal	0.151–0.261	1.015	−5.103	7.134	0.061
Superonasal	0.160–0.240	2.296	−2.778	7.370	0.642
Inferonasal	0.192–0.306	2.588	−2.076	7.253	0.540
Inferotemporal	0.194–0..304	4.823	−0.234	9.88	**0.012**

Coefficient represents the increase in the value of mfPhNR/B in each sector when GCIPL average, mRNFL average or GCC average changes by 75th percentile from 25th percentile. All multivariable regression models were adjusted for age and spherical equivalent

*mfPhNR/B* multifocal photopic negative response to multifocal B-wave ratio, *mfERG* multifocal electroretinogram, *GCIPL* Ganglion cell-inner plexiform layer, *mRNFL* Macular retinal nerve fiber layer, *GCC* Ganglion cell complex, *OCT* Optical coherence tomopraphy, *IQR* interquartile range (25th percentile – 75th percentile), *LCL* lower confidence limit, *UCL* upper confidence limit

The mfPhNR/B in the inferotemporal sector was significantly correlated with the average thickness of mRNFL, and GCC (coefficient = 2.363, *P* = 0.003 and coefficient = 4.823, *P* = 0.012, respectively).

## Discussion

This study investigated the association between the morphological statuses of the macular region measured by OCT, the functional status including two mfERG parameters with N/T and mfRhNR/B, and the sensitivities of SAP and determined the clinical superiority between mfPhNR/B and N/T in the same glaucoma patients. Better correlations between VF parameters or OCT thickness and the N/T were found, comparing with the mfRhNR/B.

In this study, the P1 component of the first slice of the second-order kernel response was elicited by the stimuli in the central 5° region in OAG patients (Fig. 2). However, we adopted the N/T instead of the P1 component because the latter boasts relatively large intersubject variations [12]. The nasal-temporal asymmetry of the mfERG scans is affected in glaucomatous eyes [11, 12]. We previously reported significant differences existed in the N/T of the first slice of the second-order kernel of the mfERG scans in the central 5° between normal and NTG eyes and found significant correlations between the N/T and the MT obtained with the HFA 30–2 and 10–2 results [11, 14], which are in agreement with the current study in glaucoma patients. The N/T was significantly correlated with the MT of HFA 10–2 in the superior and the superonasal sectors and TD in superonasal sector (Table 2). In the current study, we used not only the N/T of mfERG scans and HFA 10–2 but also OCT parameters and, furthermore, we classified the stimulus points on the HFA 10–2 as corresponding to each of six GCIPL measurement ellipse sectors into six groups (Fig. 1) based on the report of RGC displacement [16]. Although GCC thickness can predict function within the central area in eyes with glaucoma, adjusting for the RGC displacements is essential in evaluating the association between structure and function in the macula [21]. Resultantly, both relationships—between N/T and mRNFL, GCIPL, and GCC thickness—had significant correlations in the superior, superotemporal, and superonasal areas (i.e. inferior, inferonasal, and inferotemporal retina areas) (Table 2). Furthermore, the GCIPL thickness in the inferotemporal sector (i.e. superonasal retina area) significantly correlated with N/T. Correction coefficients of GCIPL and GCC thickness to N/T were higher than those of mRNFL thickness to N/T. Since N/T of mfERG is derived from RGC, GPIPL including RGC cell bodies and their dendrites or GCC including RGC cell bodies, their dendrites, and their axons might show better associations to N/T than mRNFL axons. We also found statistically significant correlations exist between the MT and N/T (Table 2) in the superior and superonasal VF sectors (i.e., inferior and inferotemporal retinal areas). These findings may be related to unique glaucomatous VF defect patterns found in the superior and superonasal areas that correspond to inferior and inferotemporal retinal damages [16]. Lee et al. reported that progressive GCIPL thinning in the temporal sector occurred faster in affected than in unaffected hemifields [16]. Na et al. found that the macula cube volume and the thicknesses of the temporal and inferior macular sectors decreased faster in progressively glaucomatous eyes [22]. These reports are in agreement with our findings.

The nasal amplitudes in the first slice of the second-order kernel of mfERG within 5° were significantly smaller than the temporal amplitudes in normal subjects [11, 12] (The average N/T ratio in the normal subjects was 0.64 with the mean age of 61.9 years [11]), whereas the difference became smaller and thus the N/T ratio became larger (approaching 1.0) after glaucoma development and progression. If the difference in amplitude became insignificant (i.e., saturated) in a very early stage of glaucoma, the ratio reached 1.0 in the early stage of glaucoma progression and, thus, the N/T was not useful clinically for monitoring glaucomatous functional change; however, the nasal amplitudes were still significantly smaller than the temporal amplitudes (3.05 nV/deg^2^ in the nasal vs. 3.92 in the temporal hemifields; *P* < 0.001, N/T = 0.81) in this study population with an average MD of − 7.00 dB, i.e., patients with moderate glaucoma. Thus, these abovementioned studies and ours suggest that glaucomatous changes are often found in the superior or superonasal regions of central VFs and that N/T of mfERG in the central 5° may be useful for detecting glaucomatous VF and the corresponding inferior or inferotemporal inner OCT changes at least until reaching the moderate stage of disease.

The PhNR amplitudes of the focal macular ERG scans can be used to assess the damages of the RGCs in glaucoma and the decrease in the PhNR amplitudes was associated with reductions in the cpRNFL and mRNFL thicknesses [6, 13]. The amplitudes of the PhNR of the focal ERG scans correlated with the corresponding cpRNFL thicknesses when measured by scanning laser polarimetry in the superotemporal and inferotemporal regions [23]. Recently, the PhNR/B was reported to exhibit the lowest magnitude of test–retest variability and to be the optimal measure of the PhNR [10]. In the current study, we measured the mfPhNR/B. Among sectorial values, significant correlation was found between the inferotemporal region of mfPhNR/B and the average GCIPL, mRNFL and GCC thickness (Table 4). The mfPhNR/B in the supeotemporal was significantly negatively correlated with GCIPL (Table 4), and the MT, and total deviation in the superotemporal VF area (i.e. inferonasal retinal area) (Table 3). The decrease in the mfPhNR/B amplitudes should be associated with reductions in the retinal thickness and sensitivities. One of the reasons for this discrepancy may be regional disagreement in the measurement areas. Better association (The coefficient of GCC was the highest) was observed between the full GCC and PhNR/B, comparing to RNFL or GCIPL. The comparison between the mfPhNR/B and OCT macular parameters is limited by the difference in spatial correspondence- the main OCT parameters cover a much larger region than the central stimuli, and a much smaller region than the stimuli in the outer annulus. Thus, better association was observed to use the full GCC rather than mRNFL and mGCIPL parameters separately, as this is generally more robust given the low resolution of the HD-OCT Macular Cube scan. Machida et al. also found that the PhNRs of focal ERG were well-correlated with the GCC thickness within the central macula [5]. Kaneko et al. used mfERG to assess the PhNR recorded from five macular retinal locations and found selective reductions in the mfERG component only present within the central 15 degrees. Thus, another possibility is that the mfPhNR/B may be most useful within the central macula because of the highest RGC density being in the macula [15]. A multifocal technique could assess multiple independent stimulus locations simultaneously; however, the best way to go about topographic analysis has not yet been elucidated [24]. Further research that the measurement areas of mfPhNR/B correspond to those of OCT to elucidate the utility of the mfPhNR/B.

In our study, correlations between VF parameters or OCT thickness and the N/T were found, especially in the inferior and inforotemporal retinal areas corresponding to superior and superonasal VF sectors. Initial glaucomatous changes tend to occur in these areas [16, 22]. However, similar results were not obtained with between mfPhNR/B and VF or OCT parameters. The N/T was also correlated with GCC and VF in more numbers of measurement areas than the mfPhNR/B in the current study. A future study modifying the stimuli and amplitudes in the central and four more peripheral area to obtain the spatial correspondence to OCT measurement will be required to evaluate the value of mfPhNR/B. OCT measurement is a fast and completely noninvasive test. VF measurement is affected by the subject’s voluntarily. For patients whose VF tests are not reliable, only OCT measurement is the tool for objective assessment in glaucoma. The best way to go about topographic analysis of mfERG parameters has not yet been proven and further study is required regarding whether mfERG parameters in conjunction with OCT measurements in the corresponding macular region may enhance diagnostic sensitivity in glaucoma. For another subjective assessment tool of OCT, mfERG will be supplemental ones in the future if further improvements are achieved.

## Conclusions

The N/T was also correlated with GCC and VF in more numbers of measurement areas than the mfPhNR/B in the current study, however, a future study modifying the stimuli and amplitudes to obtain the spatial correspondence to OCT and VF measurement will be required to evaluate the value of mfERG.

## Supplementary Information

**Additional file 1: Figure S5.** Scatter diagrams showing the associations between the N/T of mfERG, the macula thickness of OCT, and the mean thresholds and the total deviation of HFA 10–2 in each sector in glaucoma patients. N/T or NT: Nasal to temporal amplitude ratio, mfERG: multifocal electroretinogram, OCT; Optical coherence tomopraphy, HFA 10–2: Humphrey Field Analyzer Program Central 10–2, GCIPL:Ganglion cell-inner plexiform layer, Ave: Average, SN: Superonasal, IN: Inferonasal, ST: Superotemporal, IT: Inferotemporal, I: Inferior, S: Superior, mRNFL: Macular retinal nerve fiber layer, GCC: Ganglion cell complex, MD: Mean deviation, MT: Mean threshold, TD: Total deviation

**Additional file 2: Figure S6.** Scatter diagrams showing the mfPhNR/B of mfERG, the mean threshold and the total deviation of HFA 30–2, and the correlations between them in each sector in glaucoma patients. mfPhNR/B: multifocal photopic negative response to multifocal B-wave ratio, mfERG: multifocal electroretinogram, HFA 30–2: Humphrey Visual Field Analyzer Program Central 30–2

**Additional file 3: Figure S7.** Scatter diagrams showing the associations between the mfPhNR/B of mfERG in each sector and the average thickness of the GCIPL, mRNFL, and GCC of OCT in glaucoma patients. mfPhNR/B: multifocal photopic negative response to multifocal B-wave ratio, mfERG: multifocal electroretinogram, GCIPL:Ganglion cell-inner plexiform layer, mRNFL: Macular retinal nerve fiber layer, GCC: Ganglion cell complex, OCT: Optical coherence tomopraphy, Ave: Average

## Data Availability

The data used in the current study is available from the corresponding author upon request.

## Abbreviations

N/T: Nasal to temporal amplitudes ratio
mfERG: Multifocal electroretinography
PhNR: Photopic negative response
PhNR/B: Photopic negative response to B-wave ratio
GCC: Ganglion cell complex
OCT: Optical coherence tomography
VF: Visual field
MT: Mean thresholds
mfPhNR/B: Multifocal photopic negative response to B-wave ratio
RGCs: Retinal ganglion cells
ERG: Electroretinography
SAP: Standard automated perimetry
OAG: Open angle glaucoma
NTG: Normal tension glaucoma
IOPs: Intraocular pressures
POAG: Primary open-angle glaucoma
D: Diopters
VA: Visual acuity
logMAR: Logarithm of the minimum angle of resolution
HD-OCT: High-definition optical coherence tomography
RNFL: Retinal nerve fiber layer
cpRNFL: Circumpapillary retinal nerve fiber layer
GCA: Ganglion Cell Analysis
mRNFL: Macular retinal nerve fiber layer
GCIPL: Ganglion cell and inner plexiform layer
IPL: Inner plexiform layer
HFA: Humphrey Field Analyzer
HFA 30–2: Humphrey Field Analyzer with the Central 30–2 programs
HFA 10–2: Humphrey Field Analyzer with the Central 10–2 programs
VERIS: Visual Evoked Response Imaging System Science
CRT: Cathode ray tube
mfB-wave: Multifocal B-wave
MD: Mean deviation
PSD: Pattern standard deviation
