# Supplementary material for: Relationship between structural and functional changes in glaucomatous eyes: a multifocal electroretinogram study
Source: BMC Ophthalmol. 2021 Aug 21;21:305. doi: 10.1186/s12886-021-02061-8 (PMC8379802; doi:10.1186/s12886-021-02061-8)
Supplement: Supplementary file 1 — Additional file 1: Figure S5. Scatter diagrams showing the associations between the N/T of mfERG, the macula thickness of OCT, and the mean thresholds and the total deviation of HFA 10–2 in each sector in glaucoma patients. N/T or NT: Nasal to temporal amplitude ratio, mfERG: multifocal electroretinogram, OCT; Optical coherence tomopraphy, HFA 10–2: Humphrey Field Analyzer Program Central 10–2, GCIPL:Ganglion cell-inner plexiform layer, Ave: Average, SN: Superonasal, IN: Inferonasal, ST: Superotemporal, IT: Inferotemporal, I: Inferior, S: Superior, mRNFL: Macular retinal nerve fiber layer, GCC: Ganglion cell complex, MD: Mean deviation, MT: Mean threshold, TD: Total deviation [file 12886_2021_2061_MOESM1_ESM.pptx]

## Slide 1
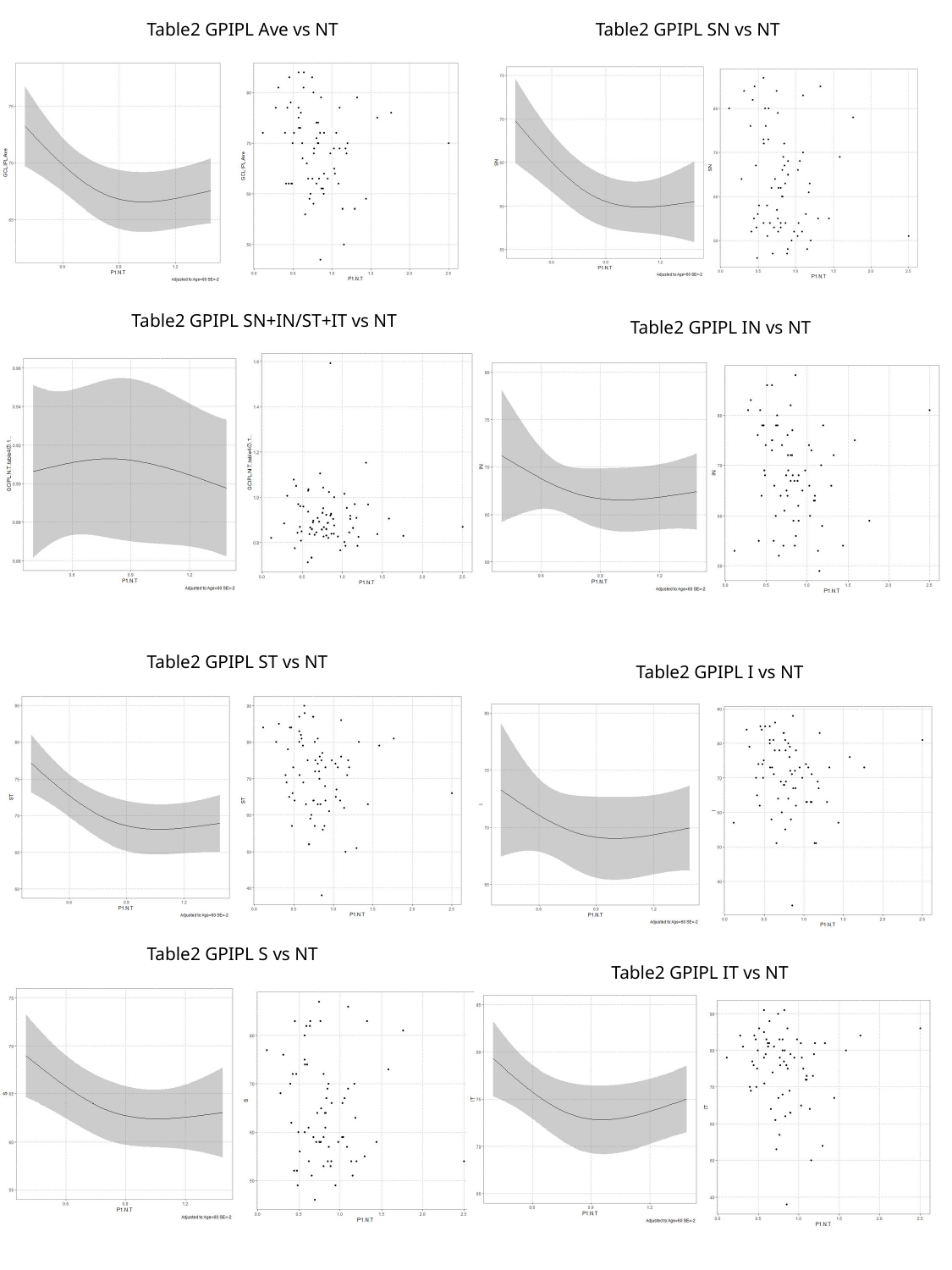

Table2 GPIPL Ave vs NT
Table2 GPIPL SN vs NT
Table2 GPIPL SN+IN/ST+IT vs NT
Table2 GPIPL IN vs NT
Table2 GPIPL ST vs NT
Table2 GPIPL I vs NT
Table2 GPIPL S vs NT
Table2 GPIPL IT vs NT

## Slide 2
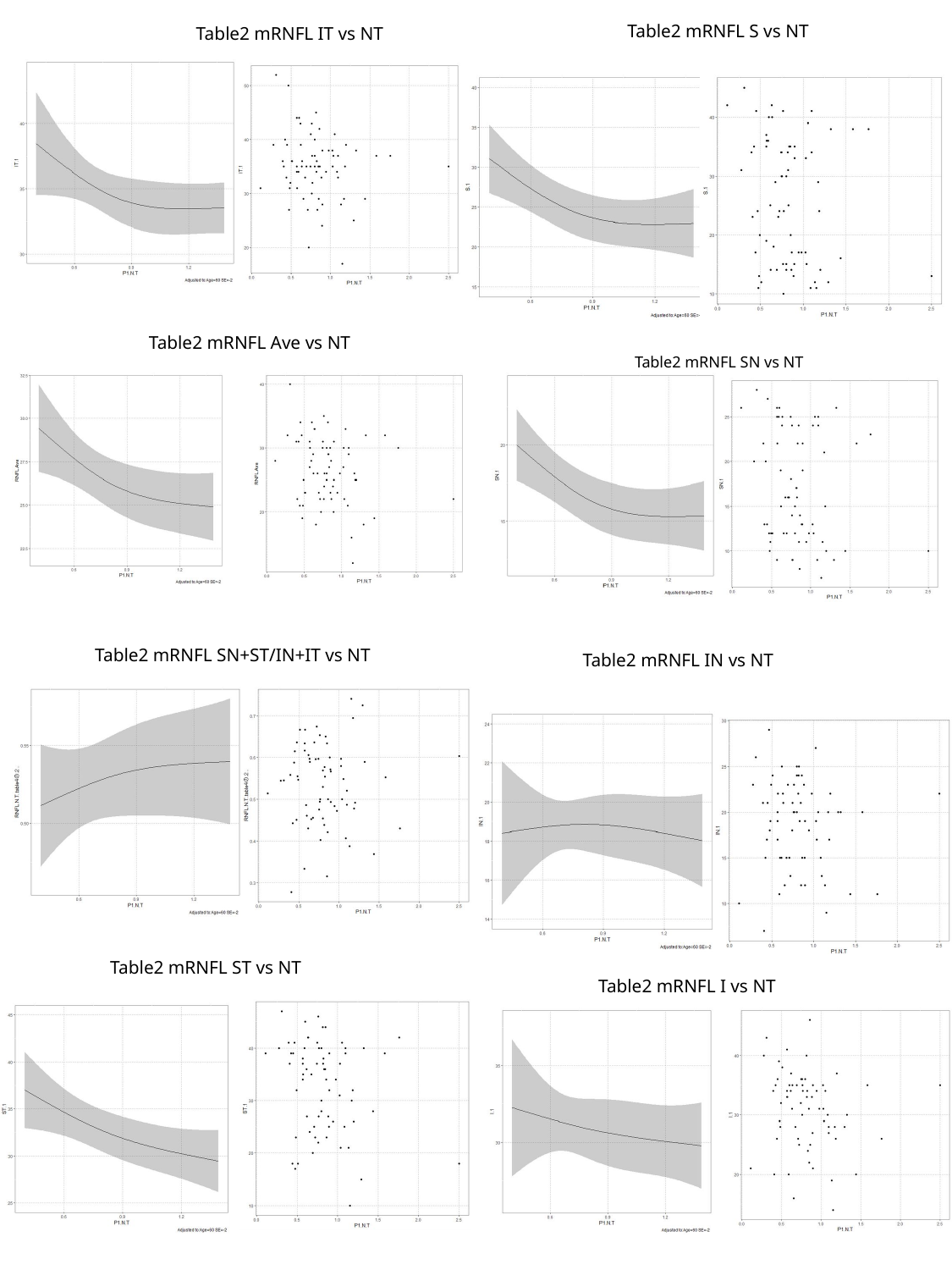

Table2 mRNFL S vs NT
Table2 mRNFL IT vs NT
Table2 mRNFL Ave vs NT
Table2 mRNFL SN vs NT
Table2 mRNFL IN vs NT
Table2 mRNFL SN+ST/IN+IT vs NT
Table2 mRNFL ST vs NT
Table2 mRNFL I vs NT

## Slide 3
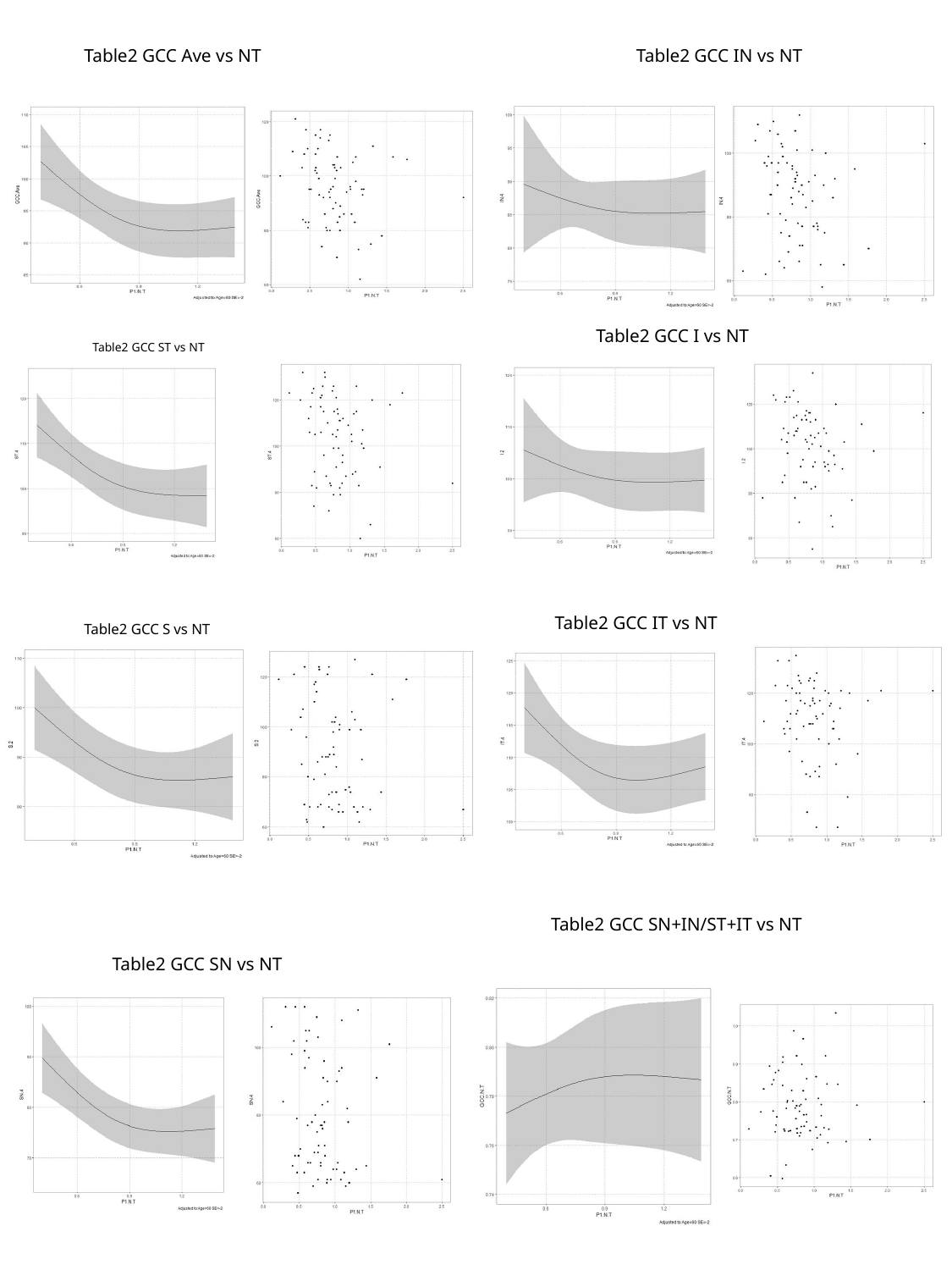

# Table2 GCC Ave vs NT
Table2 GCC IN vs NT
Table2 GCC I vs NT
Table2 GCC ST vs NT
Table2 GCC IT vs NT
Table2 GCC S vs NT
Table2 GCC SN+IN/ST+IT vs NT
Table2 GCC SN vs NT

## Slide 4
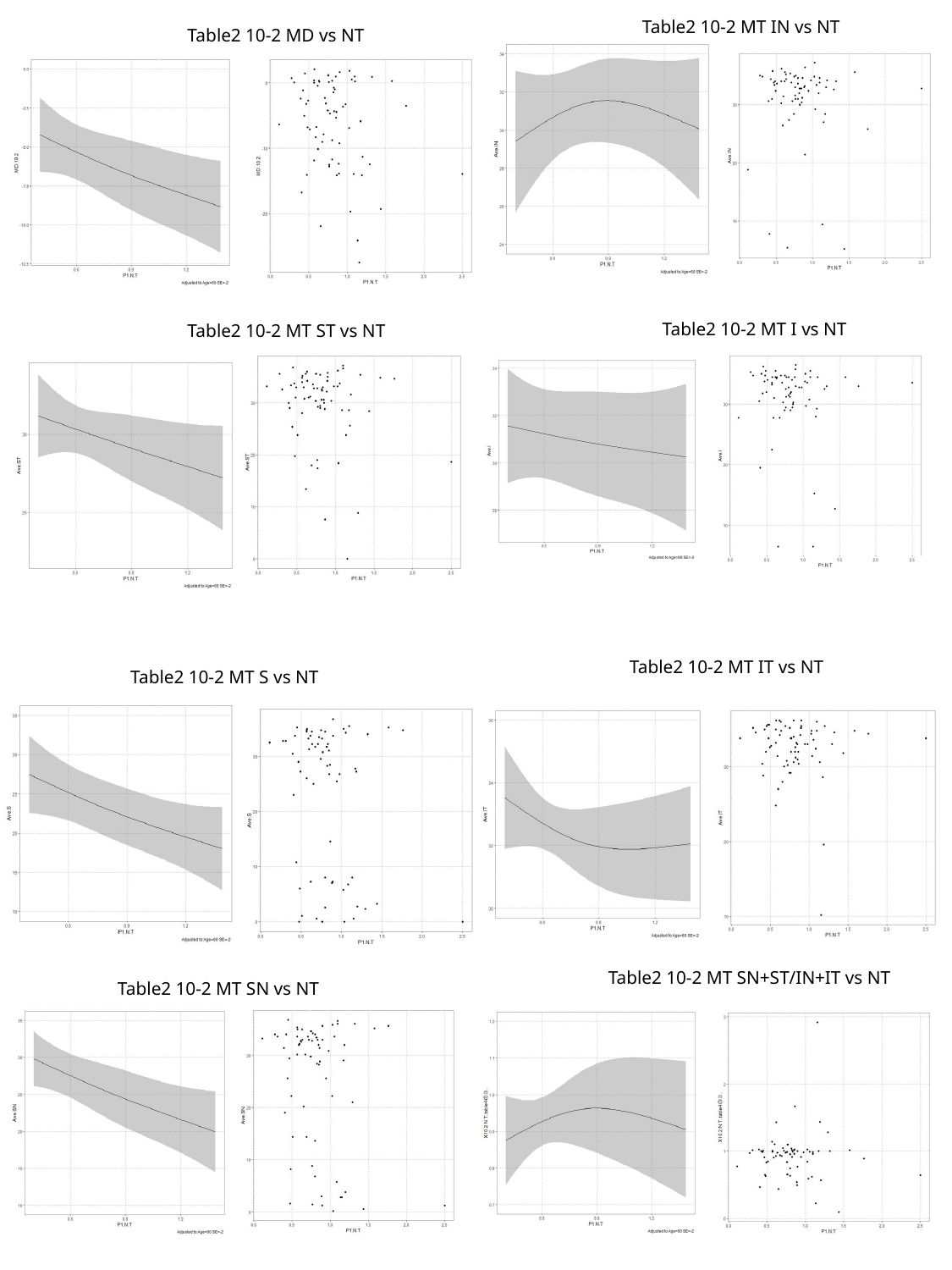

Table2 10-2 MT IN vs NT
Table2 10-2 MD vs NT
Table2 10-2 MT I vs NT
Table2 10-2 MT ST vs NT
Table2 10-2 MT IT vs NT
Table2 10-2 MT S vs NT
Table2 10-2 MT SN+ST/IN+IT vs NT
Table2 10-2 MT SN vs NT

## Slide 5
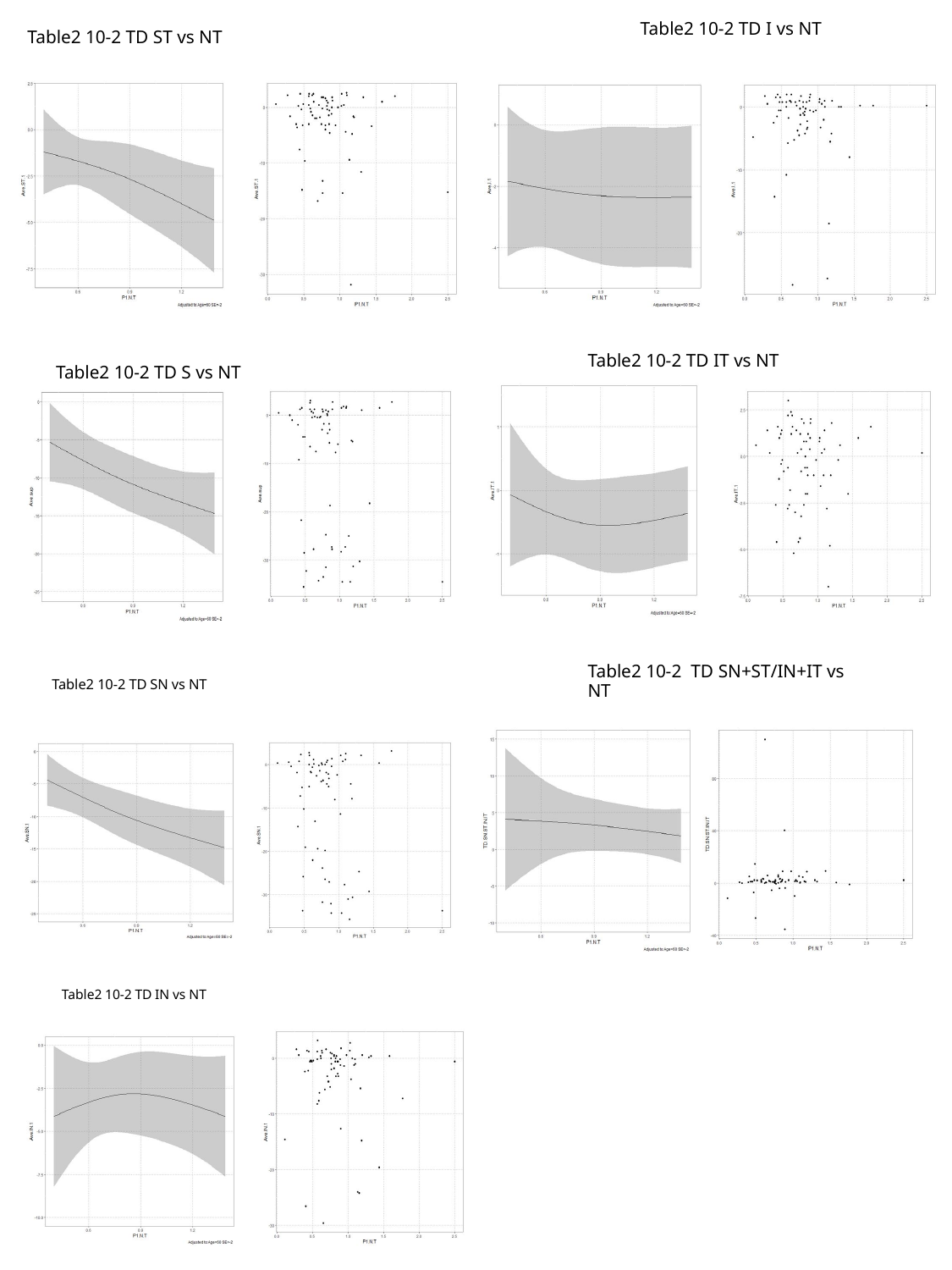

Table2 10-2 TD I vs NT
# Table2 10-2 TD ST vs NT
Table2 10-2 TD IT vs NT
Table2 10-2 TD S vs NT
Table2 10-2 TD SN+ST/IN+IT vs NT
Table2 10-2 TD SN vs NT
Table2 10-2 TD IN vs NT
