# Supplementary material for: Relationship between structural and functional changes in glaucomatous eyes: a multifocal electroretinogram study
Source: BMC Ophthalmol. 2021 Aug 21;21:305. doi: 10.1186/s12886-021-02061-8 (PMC8379802; doi:10.1186/s12886-021-02061-8)
Supplement: Supplementary file 2 — Additional file 2: Figure S6. Scatter diagrams showing the mfPhNR/B of mfERG, the mean threshold and the total deviation of HFA 30–2, and the correlations between them in each sector in glaucoma patients. mfPhNR/B: multifocal photopic negative response to multifocal B-wave ratio, mfERG: multifocal electroretinogram, HFA 30–2: Humphrey Visual Field Analyzer Program Central 30–2 [file 12886_2021_2061_MOESM2_ESM.pptx]

## Slide 1
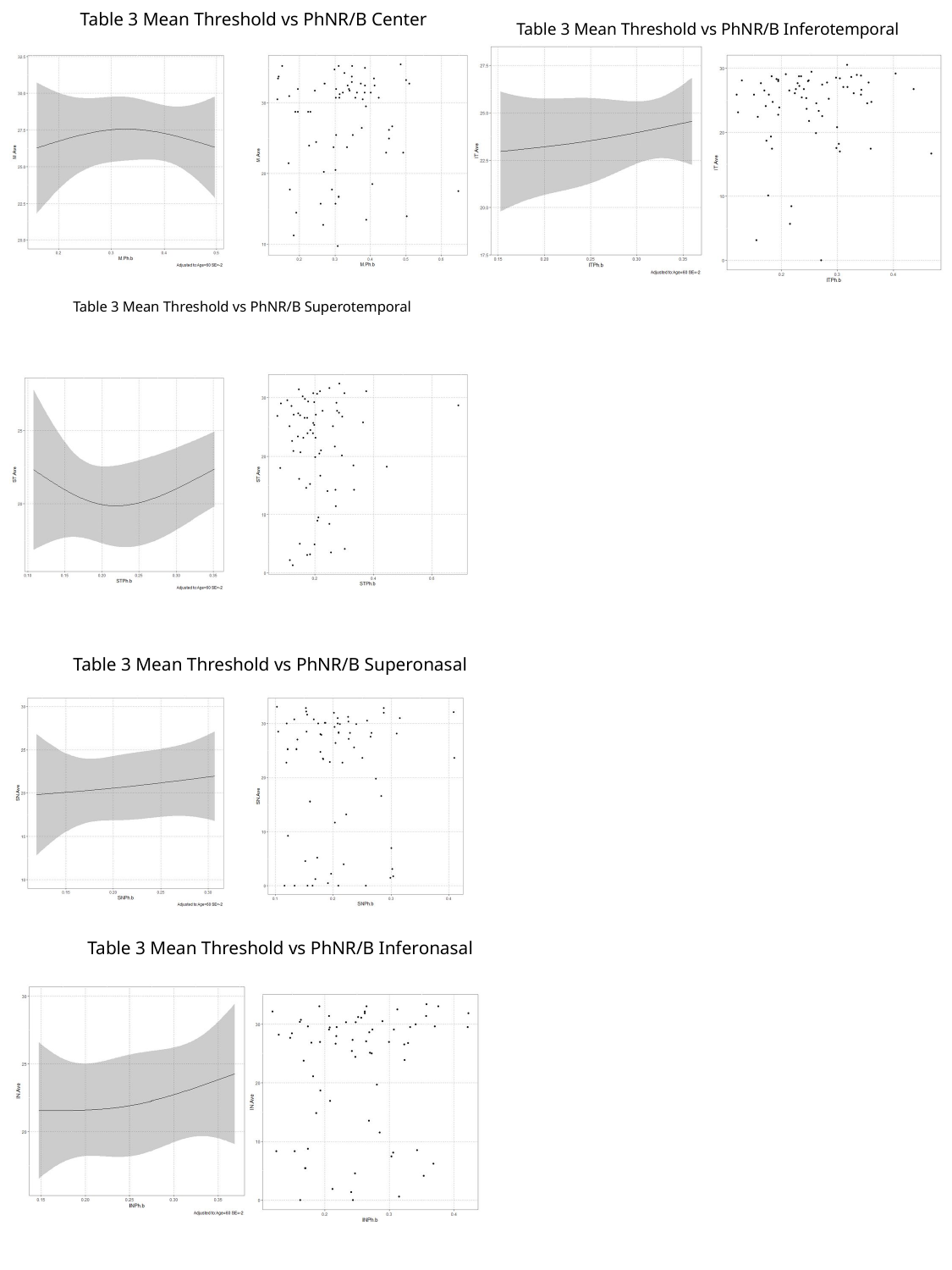

Table 3 Mean Threshold vs PhNR/B Center
Table 3 Mean Threshold vs PhNR/B Inferotemporal
Table 3 Mean Threshold vs PhNR/B Superotemporal
Table 3 Mean Threshold vs PhNR/B Superonasal
Table 3 Mean Threshold vs PhNR/B Inferonasal

## Slide 2
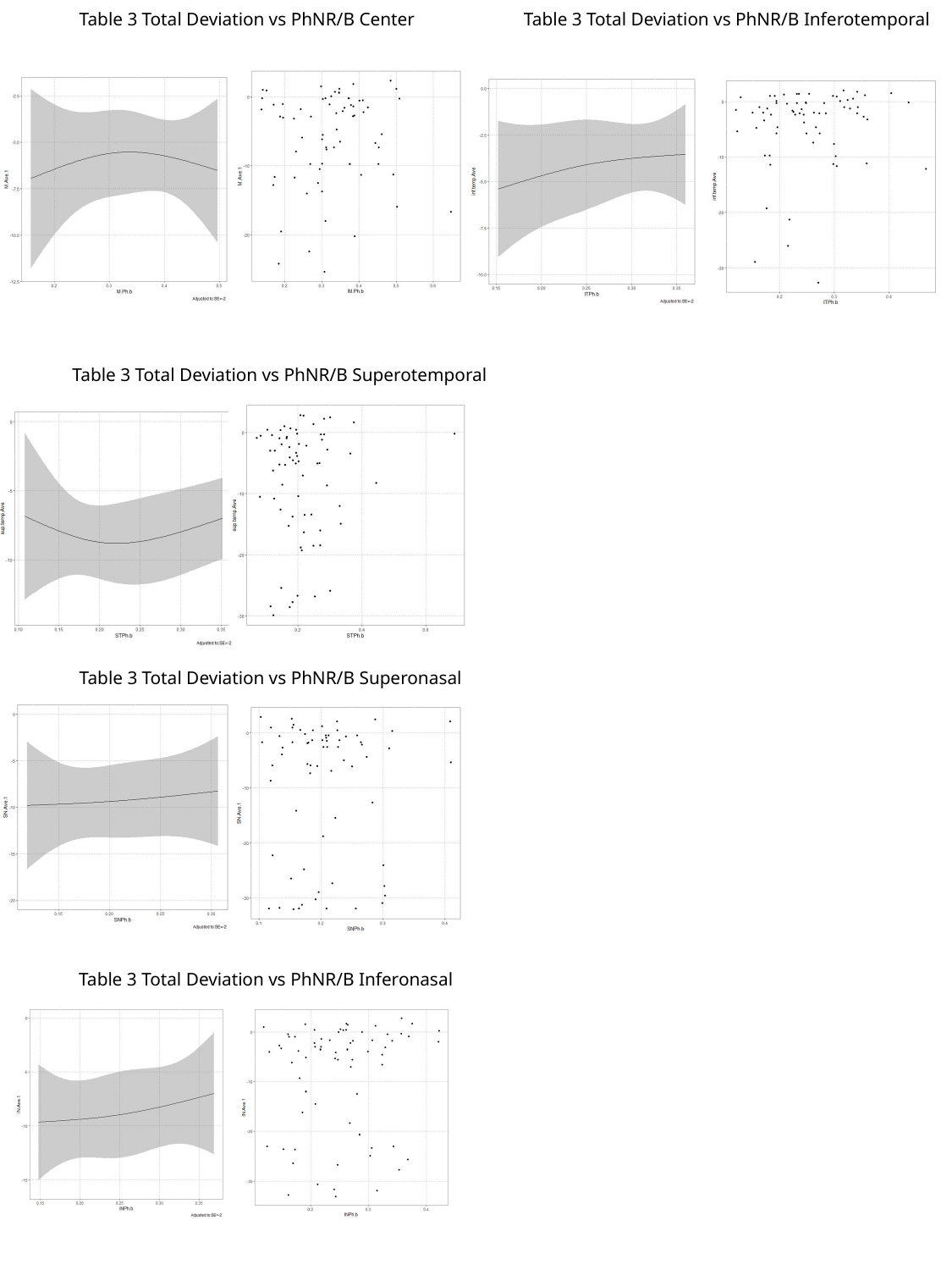

Table 3 Total Deviation vs PhNR/B Center
Table 3 Total Deviation vs PhNR/B Inferotemporal
Table 3 Total Deviation vs PhNR/B Superotemporal
Table 3 Total Deviation vs PhNR/B Superonasal
Table 3 Total Deviation vs PhNR/B Inferonasal
