# Supplementary material for: Relationship between structural and functional changes in glaucomatous eyes: a multifocal electroretinogram study
Source: BMC Ophthalmol. 2021 Aug 21;21:305. doi: 10.1186/s12886-021-02061-8 (PMC8379802; doi:10.1186/s12886-021-02061-8)
Supplement: Supplementary file 3 — Additional file 3: Figure S7. Scatter diagrams showing the associations between the mfPhNR/B of mfERG in each sector and the average thickness of the GCIPL, mRNFL, and GCC of OCT in glaucoma patients. mfPhNR/B: multifocal photopic negative response to multifocal B-wave ratio, mfERG: multifocal electroretinogram, GCIPL:Ganglion cell-inner plexiform layer, mRNFL: Macular retinal nerve fiber layer, GCC: Ganglion cell complex, OCT: Optical coherence tomopraphy, Ave: Average [file 12886_2021_2061_MOESM3_ESM.pptx]

## Slide 1
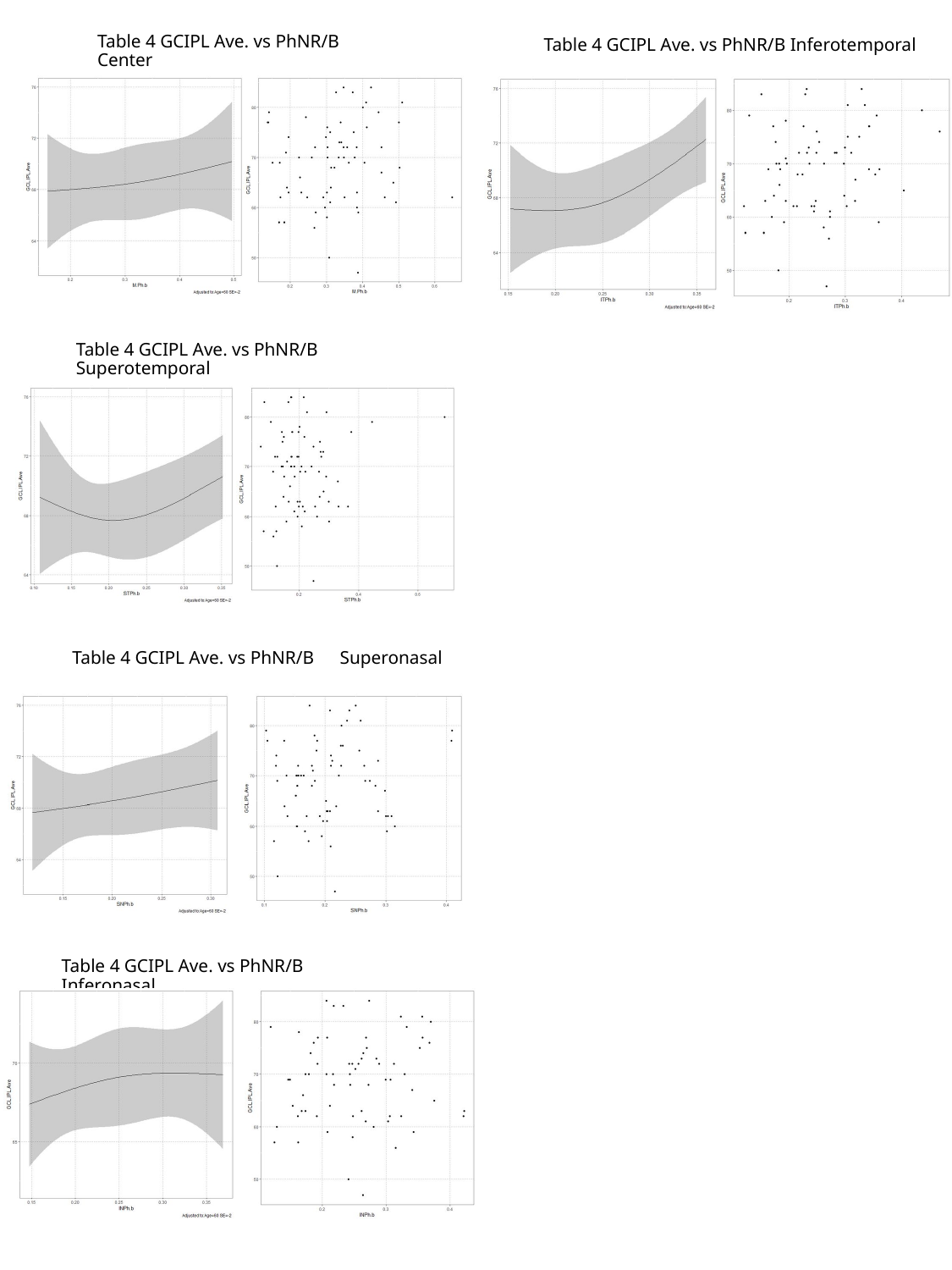

Table 4 GCIPL Ave. vs PhNR/B Center
Table 4 GCIPL Ave. vs PhNR/B Inferotemporal
Table 4 GCIPL Ave. vs PhNR/B Superotemporal
Table 4 GCIPL Ave. vs PhNR/B　Superonasal
Table 4 GCIPL Ave. vs PhNR/B Inferonasal

## Slide 2
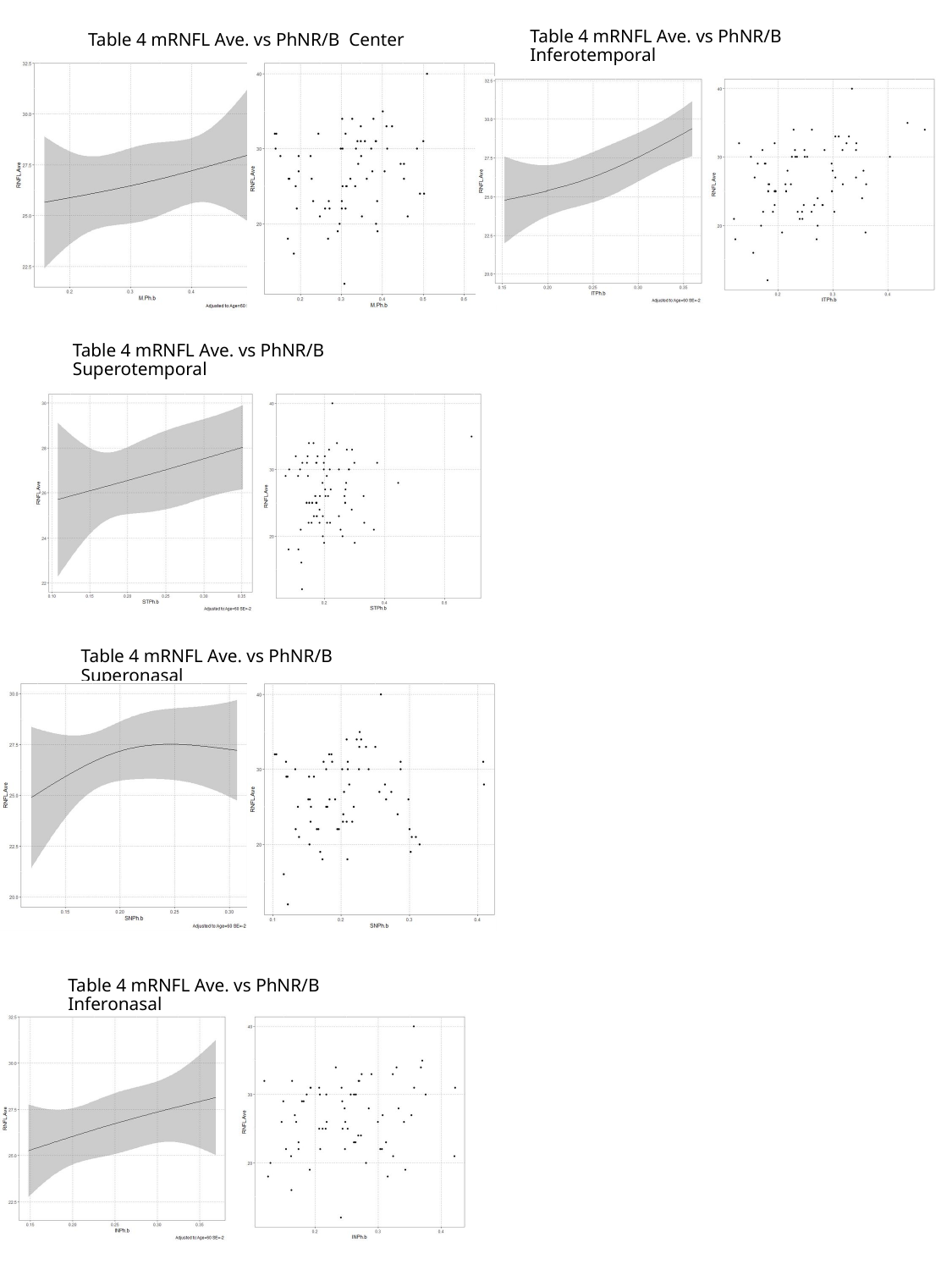

Table 4 mRNFL Ave. vs PhNR/B Inferotemporal
Table 4 mRNFL Ave. vs PhNR/B Center
Table 4 mRNFL Ave. vs PhNR/B Superotemporal
Table 4 mRNFL Ave. vs PhNR/B Superonasal
Table 4 mRNFL Ave. vs PhNR/B Inferonasal

## Slide 3
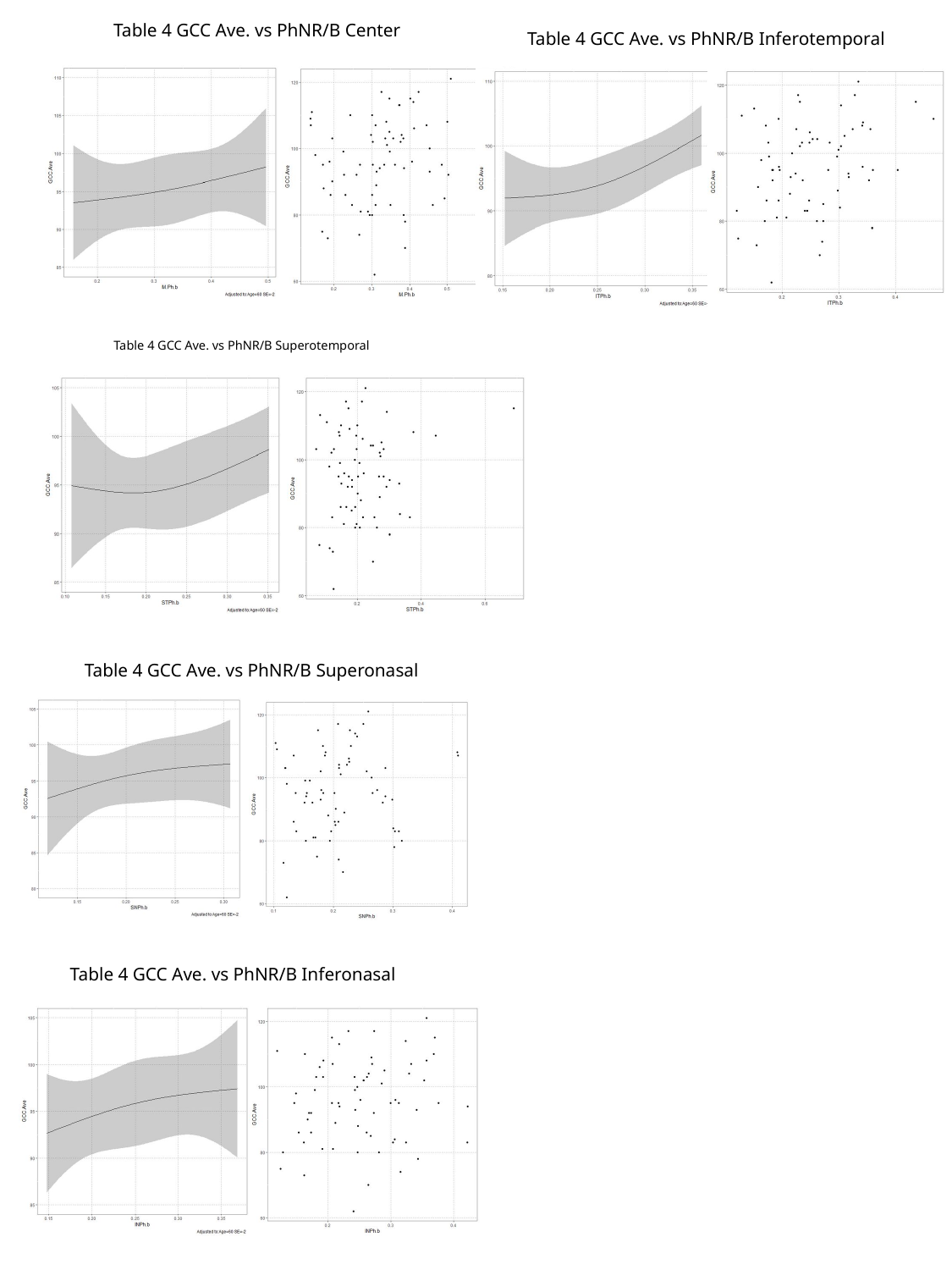

Table 4 GCC Ave. vs PhNR/B Center
Table 4 GCC Ave. vs PhNR/B Inferotemporal
Table 4 GCC Ave. vs PhNR/B Superotemporal
Table 4 GCC Ave. vs PhNR/B Superonasal
Table 4 GCC Ave. vs PhNR/B Inferonasal
